# Error Bound of Mode-Based Additive Models

**DOI:** 10.3390/e23060651

**Published:** 2021-05-22

**Authors:** Hao Deng, Jianghong Chen, Biqin Song, Zhibin Pan

**Affiliations:** 1 College of Science, Huazhong Agricultural University, Wuhan 430070, China; dengh@mail.hzau.edu.cn; 2 College of Electrical and New Energy, China Three Gorges University, Yichang 443002, China; chenjh97@126.com

**Keywords:** modal regression, additive models, reproducing kernel Hilbert spaces, error bound

## Abstract

Due to their flexibility and interpretability, additive models are powerful tools for high-dimensional mean regression and variable selection. However, the least-squares loss-based mean regression models suffer from sensitivity to non-Gaussian noises, and there is also a need to improve the model’s robustness. This paper considers the estimation and variable selection via modal regression in reproducing kernel Hilbert spaces (RKHSs). Based on the mode-induced metric and two-fold Lasso-type regularizer, we proposed a sparse modal regression algorithm and gave the excess generalization error. The experimental results demonstrated the effectiveness of the proposed model.

## 1. Introduction

Regression estimation and variable selection are two important tasks for high-dimensional data mining [1]. Sparse additive models [2,3], aiming to deal with the above tasks simultaneously, have been extensively investigated in the mean regression setting. As a class of models between linear and nonparametric regression, these methods inherit the flexibility from nonparametric regression and the interpretability from linear regression. Typical methods include COSSO [4] and SpAM [2] and its variants, such as Group SpAM [3], SAM [5], Group SAM [6], SALSA [7], MAM [8], SSAM [9], and ramp-SAM [10]. From the lens of nonparametric regression, the additive structure on the hypothesis space is crucial to overcome the curse of dimensionality [7,11,12].

Usually, the aforementioned models are limited to the estimation of the conditional mean under the mean-squared error (MSE) criterion. However, for the complex non-Gaussian noises (e.g., the skewed noise, the heavy-tailed noise), it is difficult to extract the intrinsic trends from the mean-based approaches, resulting in degraded performance. Beyond the traditional mean regression, it is interesting to formulate a new regression framework under the (conditional) mode-based criterion. With the help of the recent works in [13,14,15,16,17,18,19], this paper aimed to propose a new robust sparse additive model, rooted in modal regression associated with the RKHS.

As an alternative approach to mean regression, modal regression has been investigated on statistical behavior [14,15,17] and real-world applications [20,21]. Yao [14] proposed a modal linear regression algorithm and characterized its theoretical properties under the global mode assumption. As a natural extension of Lasso [22], Wang et al. [15] considered the regularized modal regression and established its analysis on the generalization bound and variable selection consistency. Feng et al. [17] studied modal regression by a learning theory approach and illustrated its relation with MCC [23,24]. Different from the above global approaches, some local modal regression algorithms were formulated in [16,25] with convergence guarantees. Recent literature [26] gave a general overview of modal regression, and a more comprehensive list of references can be found there.

The proposed robust additive models are formulated under the Tikhonov regularization scheme, which involves three building blocks, including the mode-based metric, the RKHS-based hypothesis space, and two Lasso-type penalties. Since the linear function space, polynomial function space, and Sobolev/Besov space are special cases of the RKHS, the kernel-based function space is more flexible than the traditional spline-based spaces or other dictionary-based hypotheses [2,5,27,28,29]. The mode-induced regression metric is robust to the non-Gaussian noise according to the theoretical and empirical evaluations [14,15,17]. The regularized penalty addresses the sparsity and smoothness of the estimator, which has shown promising performance for mean regression [2,29,30,31]. Therefore, different from mean-based kernel regression and additive models, the mode-based approach enjoys robustness and interpretability simultaneously due to its metric criterion and trade-off penalty. The estimator of our approach can be obtained by integrating the half-quadratic (HQ) optimization [32] and the second-order cone programming (SOCP) [33].

The rest of this article is organized as follows. After introducing the robust additive model in Section 2, we state its generalization error bound in Section 3. Finally, Section 5 ends this paper with a brief conclusion.

## 2. Methodology

### 2.1. Modal Regression

In this section, we recall the basic background on modal regression [19,34]. Let X be a compact subset of $R^{p}$ associated with the input covariate vector and Y∈R be the response variable set. In this paper, we considered the following nonparametric model:

(1) $$Y=f^{*}\left(X\right)+\epsilon ,$$

where $X={\left(X_{1},\ldots ,X_{p}\right)}^{T}\in X$, Y∈Y, and ϵ is a random noise. For feasibility, we denote by ρ the underlying joint distribution of (X,Y) generated by (1).

Being different from the traditional mean regression under the noise condition E(ϵ|X=x)=0 (e.g., Gaussian noise), we just require that the mode of the conditional distribution of ϵ equal zero at each x∈X. That is:

(2) $$\forall x\in X,mode\left(\epsilon |X=x\right)=\arg\max_{t\in R}P_{\epsilon |X}\left(t|X=x\right)=0,$$

where $P_{\epsilon |X}$ is the conditional density of ϵ given *X*. Notice that the zero condition is not specified to the homogeneity or symmetry distribution of noise ϵ, and some non-Gaussian noises (e.g., the skewed noise, the heavy-tailed noise) are not excluded.

From (1), we further deduce that:

$$\begin{array}{ccc} f^{*}\left(u\right) & := & \sum_{j=1}^{p}f_{j}^{*}\left(u_{j}\right)=mode\left(Y|X=u\right)=\arg\max_{t}P_{Y|X}\left(t|X=u\right), \end{array}$$

where $u={\left(u_{1},\ldots ,u_{p}\right)}^{T}\in X$ and $P_{Y|X}$ denotes the density of *Y* conditional on *X*. Then, the purpose of modal regression is to find the target function $f^{*}$ according to the empirical data $z={\left\{z_{i}\right\}}_{i=1}^{n}={\left\{\left(x_{i},y_{i}\right)\right\}}_{i=1}^{n}$ drawn independently from ρ.

For modal regression, the performance of a predictor f:X→R is measured by the mode-based metric:

(3) $$R\left(f\right)=\int_{X}P_{Y|X}\left(f\left(x\right)|X=x\right)d\rho_{X}\left(x\right),$$

where $\rho_{X}$ is the marginal distribution of ρ with respect to input space X.

Although the target function $f^{*}$ is the maximizer of R(f) over all measurable functions, it cannot be estimated directly via maximizing (3) due to the unknown $P_{Y|X}$ and $\rho_{X}$. Fortunately, some indirect density-estimation-based strategies were proposed in [14,15,17]. As shown in Theorem 5 of [17], R(f) equals the density function of random variable $E_{f}=Y-f\left(X\right)$ at zero, e.g.,

$$R\left(f\right)=P_{E_{f}}\left(0\right).$$

Therefore, we can find an approximation of $f^{*}$ by maximizing the empirical version of $P_{E_{f}}\left(0\right)$ with the help of kernel density estimation (KDE).

Let $K_{\sigma }:R\times R\to R_{+}$ be a kernel with bandwidth σ, and its representing function ϕ:R→[0,∞) satisfies $\phi \left(\frac{u-u^{\prime }}{\sigma }\right)=K_{\sigma }\left(u,u^{\prime }\right),\forall u,u^{\prime }\in R$. Typical kernels used in KDE include the Gaussian kernel, the Epanechnikov kernel, the logistic kernel, and the sigmoid kernel. The KDE-based estimator of $P_{E_{f}}\left(0\right)$ is defined as:

$$\begin{array}{c} {\hat{P}}_{E_{f}}\left(0\right)=\frac{1}{n\sigma }\sum_{i=1}^{n}K_{\sigma }\left(y_{i}-f\left(x_{i}\right),0\right)=\frac{1}{n\sigma }\sum_{i=1}^{n}\phi \left(\frac{y_{i}-f\left(x_{i}\right)}{\sigma }\right):={\hat{R}}^{\sigma }\left(f\right). \end{array}$$

Learning models for modal regression are usually formulated by Tikhonov regularization schemes associated with the empirical metric ${\hat{R}}^{\sigma }\left(f\right)$; see, e.g., [15,35].

Naturally, the data-free modal regression metric, w.r.t.${\hat{R}}^{\sigma }\left(f\right)$, can be defined as:

$$R^{\sigma }\left(f\right)=\frac{1}{\sigma }\int_{X\times Y}\phi \left(\frac{y-f(x)}{\sigma }\right)d\rho \left(x,y\right).$$

In theory, the learning performance of estimator f:X→R can be evaluated in terms of $R\left(f\right)-R\left(f^{*}\right)$, which can be further bounded via $R^{\sigma }\left(f\right)-R^{\sigma }\left(f^{*}\right)$ (see Theorem 10 in [17]).

**Remark** **1.** *As illustrated in [17], when taking $K_{\sigma }$ as a Gaussian kernel, the modal regression for maximizing $R^{\sigma }\left(f\right)$ is consistent with learning under the maximum correntropy criterion (MCC). By employing different kernels, we can provide rich evaluated metrics for better robust estimation.*

### 2.2. Mode-Based Sparse Additive Models

The additive model is formulated as follows,

(4) $$Y=\sum_{j=1}^{p}f_{j}^{*}\left(X_{j}\right)+\epsilon ,$$

where $X_{j}\in X$, (j=1,2,···,p), Y∈Y, and $f_{j}^{*}$ are unknown component functions. By employing nonlinear hypothesis function spaces with an additive structure, the additive model provides better flexibility for regression estimation and variable selection [19]. In [28], the theoretical properties of the sparse additive model with the quantile loss function were discussed. We introduce some basic notation and assumptions in a similar way.

Suppose that $Ef_{j}^{*}\left(X_{j}\right)=0$ and $∥f_{j}^{*}{∥}_{K_{j}}\leq 1$ for each $f_{j}^{*}$ in (4) with j∈S. Here, $f_{j}^{*}:X_{j}\to R$ is an unknown univariate function in a reproducing kernel Hilbert space (RKHS) $H_{j}:=H_{K_{j}}$ associated with kernel $K_{j}$ and norm ${∥\cdot ∥}_{K_{j}}$ [30,31], and S⊆{1,…,p} is an intrinsic subset with cardinality |S|<p. This means each observation $\left(x_{j},y_{j}\right)$ is generated according to:

$$y_{i}=\sum_{j\in S}f_{j}^{*}\left(x_{ij}\right)+\epsilon_{i},i=1,\ldots ,n,$$

where $x_{i}={\left(x_{i1},\ldots ,x_{ip}\right)}^{T}\in R^{p}$, $f_{j}^{*}\in H_{j}$ and ϵ satisfies the condition (2).

For any given j∈{1,…,p}, denote $B_{r}\left(H_{j}\right)=\{g\in H_{j}{:∥g∥}_{K_{j}}\leq r\}$. The hypothesis space considered here is defined by:

(5) $$F=\{f=\sum_{j=1}^{p}f_{j}:f_{j}\in B_{r}\left(H_{j}\right),i=1,\ldots ,p\},$$

which is a subset of the RKHS $H=\{f=\sum_{j=1}^{p}f_{j}:f_{j}\in H_{j}\}$ with the norm:

$${∥f∥}_{K}^{2}=\inf\{\sum_{j=1}^{p}∥f_{j}{∥}_{K_{j}}^{2}:f=\sum_{j=1}^{p}f_{j}\}.$$

For each $X_{j}$ and the corresponding marginal distribution $\rho_{X_{j}}$, we denote $∥f_{j}{∥}_{2}^{2}:=\int_{X_{j}}{\left|f_{j}\left(u\right)\right|}^{2}d\rho_{X_{j}}\left(u\right)$. Given inputs ${\left\{x_{i}\right\}}_{i=1}^{n}$, define the empirical norm of each $f_{j}$ as:

$$∥f_{j}{∥}_{n}^{2}:=\frac{1}{n}\sum_{i=1}^{n}f_{j}^{2}\left(x_{ij}\right),\forall f_{j}\in H_{j},j\in \left\{1,\ldots ,p\right\}.$$

With the help of the mode-based metric (3) and the hypothesis space (5), we formulated the mode-based sparse additive model as:

(6) $$\hat{f}=\arg\max_{f\in F}\{{\hat{R}}^{\sigma }\left(f\right)-\lambda_{1}\sum_{j=1}^{p}∥f_{j}{∥}_{n}-\lambda_{2}\sum_{j=1}^{p}∥f_{j}{∥}_{K_{j}}\},$$

where $\left(\lambda_{1},\lambda_{2}\right)$ is a pair of positive regularization. The first regularization term is sparsity-promoting [11,36], and the second one guarantees smoothness in the solution.

By the representer theorem of kernel methods (e.g., [37]), the solution of (6) admits the following form:

$$\hat{f}\left(u\right)=\sum_{i=1}^{n}\sum_{j=1}^{p}{\hat{\alpha }}_{ij}K\left(u_{j},x_{ij}\right),u={\left(u_{1},\ldots ,u_{p}\right)}^{T}$$

with a collection of coefficients $\left\{{\hat{\alpha }}_{j}={\left(\alpha_{1j},\ldots ,\alpha_{nj}\right)}^{T}\in R^{n}:j=1,\ldots ,p\right\}$.

The optimal coefficients with respect to (6) are the solution to the following non-convex optimization:

$$\begin{array}{c} \max_{\alpha_{j}\in R^{n},\alpha_{j}^{T}K_{j}\alpha_{j}\leq 1}\{\frac{1}{n}\sum_{i=1}^{n}\phi \left(\frac{y_{i}-\sum_{j=1}^{p}K_{ji}^{T}\alpha_{j}}{\sigma }\right)-\frac{\lambda_{1}}{\sqrt{n}}\sum_{j=1}^{p}∥K_{j}\alpha_{j}{∥}_{2}-\lambda_{2}\sum_{j=1}^{p}\sqrt{\alpha_{j}^{T}K_{j}\alpha_{j}}\} \end{array}$$

where $K_{ji}={\left(K_{j}\left(x_{1j},x_{ij}\right),\ldots ,K_{j}\left(x_{nj},x_{ij}\right)\right)}^{T}\in R^{n}$ and $K_{j}={\left(K_{j}\left(x_{ij},x_{lj}\right)\right)}_{i,l}^{n}=\left(K_{j1},\ldots ,K_{jn}\right)\in R^{n\times n}$.

**Remark** **2.** *There are various combinations of sparsity and smoothness regularization for additive models [2,3,29,30,31]. The regularization in this paper adopting a two-fold group Lasso scheme, which was employed in [28], but in quantile regression settings, is also different from the coefficient-based regularized modal regression in [19].*

**Remark** **3.** *From the lens of computation, the proposed algorithm (6) can be transformed into a regularized least-squares regression problem by HQ optimization [32]. Then, the transformed problem can be tackled with the SOCP [33] easily.*

## 3. Error Analysis

This section states the upper bounds of the excess quantity $R\left(f^{*}\right)-R\left(\hat{f}\right)$. For the ease of presentation, we only considered the special setting where $H_{j}\equiv H_{j^{\prime }},\forall j,j^{\prime }\in \left\{1,\ldots ,p\right\}$, and we denote ${⊕}_{j=1}^{p}H_{j}$ as $H_{K}$ with supK(x,x)≤1.

Recall that the Mercer kernel K:X×X→R admits the following spectral expansion [38]:

$$K\left(x,x^{\prime }\right)=\sum_{\ell \geq 1}b_{\ell }\psi_{\ell }\left(x\right)\psi_{\ell }\left(x^{\prime }\right),x,x^{\prime }\in X,$$

where ${\left\{\left(b_{\ell },\psi_{\ell }\right)\right\}}_{\ell \geq 1}$ are the pairs of eigenvalue-eigenfunctions of integral operator $Tf:\int K\left(\cdot ,x\right)f\left(x\right)d\rho_{X}\left(x\right)$ with $b_{1}\geq b_{2}\geq \ldots \geq 0$.

To evaluate the complexity of $H_{K}$ in terms of the decay rate of eigenvalues ${\left\{b_{\ell }\right\}}_{\ell \geq 1}$ [27,28], we refer to Assumption 1 in [28] as the basis of our analysis.

**Assumption** **1.** *There exist s∈(0,1) and constant $c_{1}>0$ such that $b_{\ell }\leq c_{1}\ell^{-\frac{1}{s}}$, ∀ℓ≥1.*

As illustrated in [27,28], the requirement s<1 is a weak condition since $\sum_{\ell }b_{\ell }=EK\left(x,x\right)\leq 1$. In particular, it holds $b_{\ell }≍\ell^{-2h}$ for the Sobolev space $H_{K}=W_{2}^{h}\left(h>\frac{1}{2}\right)$ with the Lebesgue measure on [0,1].

To describe the hypothesis in RKHS, we refer to Assumption 2 in [28].

**Assumption** **2.** *For some s∈(0,1) given in Assumption 1, there exists a positive constant $c_{2}$ such that ${∥f∥}_{\infty }\leq c_{2}{∥f∥}_{2}^{1-s}{∥f∥}_{K}^{s}$, $\forall f\in H_{K}$.*

**Remark** **4.** *To understand the statistical performance of the proposed estimator without any “correlatedness” conditions on covariates, Rademacher complexity [39] was used to measure functional complexity in [28]. We drew on the experience of [28].*

In general, Assumption 2 is stronger than Assumption 1 and is satisfied when the RKHS is continuously embeddable in a Sobolev space. For the uniformly bounded ${\left\{\psi_{\ell }\right\}}_{\ell \geq 1}$, this sub-norm condition is consistent with Assumption 1.

For any given independent input variables ${\left\{x_{i}\right\}}_{i=1}^{n}\subset X$, define the Rademacher complexity:

$$R_{n}\left(f\right):=\frac{1}{n}\sum_{i=1}^{n}\sigma_{i}f\left(x_{i}\right),\forall f\in H_{K},$$

where ${\left\{\sigma_{i}\right\}}_{i=1}^{n}$ is an i.i.d. sequence of Rademacher variables that take {±1} with probability 1/2. As shown in [40], it holds:

$$ER_{n}\{f\in H_{K}{\{∥f∥}_{K}={1,∥f∥}_{2}\leq t\}\}≍\frac{1}{\sqrt{n}}{\left[\sum_{\ell }^{\infty }\min\left\{t^{2},b_{\ell }\right\}\right]}^{\frac{1}{2}}.$$

Moreover, from Assumption 1, define:

$$\begin{array}{ccc} \gamma_{n} & := & \inf\{\gamma \geq \sqrt{\frac{A\log\tilde{p}}{n}},E[\underset{{∥f∥}_{K}=1,{∥f∥}_{2}\leq t}{\sup}|R_{n}\left(f\right)|]\leq \gamma t+\gamma^{2},\forall t\in \left(0,1\right)\}\\ & ≍ & \max\{\sqrt{\frac{A\log\tilde{p}}{n}},{\left(\frac{1}{n}\right)}^{\frac{1}{2(1+\alpha )}}\}. \end{array}$$

The main idea of our error analysis is to first state a theory result for a defined event and then investigate the behavior of $\hat{f}$ in (6) conditional on that event.

Define $\eta \left(t\right):=\max\left\{1,\sqrt{t},t/\sqrt{n}\right\}$ for any t>0 and $\xi_{n}:=\xi_{n}\left(\lambda \right)=\max\left\{\lambda^{-\frac{\alpha }{2}}n^{-\frac{1}{2}},\lambda^{-\frac{1}{2}}n^{-\frac{1}{1+\alpha }},\sqrt{\frac{\log p}{n}}\right\}$, and consider the event:

$$\begin{array}{ccc} \theta (t) & = & \left\{\right|\frac{1}{n}\sum_{i=1}^{n}\epsilon_{i}f\left(x_{i}\right)|\leq c_{\alpha }\eta \left(t\right)\xi_{n}{(∥f∥}_{2}+\lambda^{\frac{1}{2}}{∥f∥}_{K}),\forall f\in H_{K}\}, \end{array}$$

where ${\left\{\epsilon_{i}\right\}}_{i=1}^{n}$ are zero-mean i.i.d. random variables with $|\epsilon_{i}|\leq L$ and $c_{\alpha }$ is a constant depending on α and *L*.

**Remark** **5.** *To analyze the behavior of the regularized estimator conditioned on the event, several basic facts of the empirical processes were introduced in [28]. Our work can be boiled down to this framework. We introduced the relevant lemmas in [28] as a stepping stone.*

**Lemma** **1.** *Let Assumptions 1 and 2 be true. If $\frac{\log p}{\sqrt{n}}\leq 1$, it holds:*

P(θ(t))≥1−exp{−t},∀λ>0,t≥1.

The following lemma (see also Theorem 4 in [41]) demonstrates the relationship between the empirical norm ${∥\cdot ∥}_{n}$ and ${∥\cdot ∥}_{2}$ for functions in $H_{K}$.

**Lemma** **2.** *For A≥1 and any given $\tilde{p}\geq p$ with $\log\tilde{p}\geq 2\log\log n$, there exists a constant c such that:*

$${∥f∥}_{2}\leq {c(∥f∥}_{n}+\gamma_{n}{∥f∥}_{K})$$

*and:*

$${∥f∥}_{n}\leq {c(∥f∥}_{2}+\gamma_{n}{∥f∥}_{K})$$

*with confidence at least $1-{\tilde{p}}^{-A}$, where $\gamma_{n}≍\max\left(\sqrt{\frac{A\log\tilde{p}}{n}},{\left(\frac{1}{n}\right)}^{\frac{1}{2(1+\alpha )}}\right)$.*

**Lemma** **3.** *Let ${\left\{z_{i}\right\}}_{i=1}^{n}\subset Z$ be independent random variables, and let Γ be a class of real-valued functions on Z satisfying:*

$$∥\gamma ∥\leq \eta_{n},\forall \gamma \in \Gamma ,\;\;\;\;\;\;and\;\;\;\;\;\;\frac{1}{n}\sum_{i=1}^{n}var\left(\gamma \left(z_{i}\right)\right)\leq \iota_{n}^{2},$$

*for some positive constants $\eta_{n}$ and $\iota_{n}$. Define $\zeta :=\sup_{\gamma \in \Gamma }\left|\frac{1}{n}\sum_{i=1}^{n}\gamma \left(z_{i}\right)-E\gamma \left(z\right)\right|$. Then,*

$$\begin{array}{c} P\{\zeta \geq E\zeta +t\sqrt{2(\iota_{n}^{2}+2\eta_{n}Ez)}+\frac{2\eta_{n}t^{2}}{3}\leq \exp\left\{-nt^{2}\right\} \end{array}$$

For any given $\Delta_{-}$ and $\Delta_{+}$, define:

$$\begin{array}{ccc} F(\Delta_{-},\Delta_{+}) & = & \{f=\sum_{j=1}^{p}f_{j}\in H_{K}:\gamma_{n}\sum_{j=1}^{p}∥f_{j}-f_{j}^{*}{∥}_{2}\leq \Delta_{-},\gamma_{n}^{2}\sum_{j=1}^{p}∥f_{j}-f_{j}^{*}{∥}_{K}\leq \Delta_{+}\}, \end{array}$$

**Lemma** **4.** *Let Assumptions 1 and 2 be true for each $H_{j}$. For any given A≥2, with confidence at least $1-{\tilde{p}}^{-A}$, it holds:*

$$\begin{array}{cc} & R^{\sigma }\left(f^{*}\right)-R^{\sigma }\left(f\right)-\left({\hat{R}}^{\sigma }\left(f^{*}\right)-{\hat{R}}^{\sigma }\left(f\right)\right)\leq c_{*}\eta \left(t_{0}\right)\left(\Delta_{-}+\Delta_{+}\right)+\exp\left\{-\tilde{p}\right\}, \end{array}$$

for any $f\in F(\Delta_{-},\Delta_{+})$ with $max\left\{\Delta_{-},\Delta_{+}\right\}\leq e^{\tilde{p}}$, where $t_{0}=2\log\left(\frac{2\sqrt{3}}{\log2}\right)+A\log\tilde{p}+2\log\tilde{p}$, $\lambda =n^{-\frac{1}{1+\alpha }}$, and $c_{*}$ is a positive constant.

**Proof.**  Denote $\Gamma =\{\gamma \left(z\right):\gamma \left(z\right)=\frac{1}{\sigma }\phi \left(\frac{y-f^{*}\left(x\right)}{\sigma }\right)-\frac{1}{\sigma }\phi \left(\frac{y-f(x)}{\sigma }\right),f\in F\left(\Delta_{-},\Delta_{+}\right)\}$. It is easy to verify that:

$$E\gamma \left(z\right)-\frac{1}{n}\sum_{i=1}^{n}\gamma \left(z_{i}\right)=R\left(f^{*}\right)-R\left(f\right)-\left(\hat{R}\left(f^{*}\right)-\hat{R}\left(f\right)\right),\gamma \in \Gamma .$$

Let $\zeta :=\sup_{\gamma \in \Gamma }\left|\frac{1}{n}\sum_{i=1}^{n}\gamma \left(z_{i}\right)-E\gamma \left(z\right)\right|$. From Lemma 3, we have:

(7) $$\zeta \leq E\zeta +\sqrt{\frac{2t(\iota_{n}^{2}+2\eta_{n}E\zeta )}{n}}+\frac{2\eta_{n}t}{3n},$$

with probability at least 1−exp{−t}, where constants $\sup_{\gamma \in \Gamma }{∥\gamma ∥}_{\infty }=\eta_{n}$ and $\sup_{\gamma \in \Gamma }\sqrt{\frac{1}{n}\sum_{i=1}^{n}var\left(\gamma \left(z_{i}\right)\right)}=\iota_{n}$. Observing that:

(8) $$\begin{array}{c} \sqrt{\frac{2t(\iota_{n}^{2}+2\eta_{n}E\zeta )}{n}}\leq \sqrt{\frac{2t\iota_{n}^{2}}{n}}+2\sqrt{\frac{\eta_{n}E\zeta }{n}}\leq \sqrt{\frac{2t}{n}}\iota_{n}+E\zeta +\frac{\eta_{n}}{n}, \end{array}$$

we can take:

(9) $$\begin{array}{c} \iota_{n}^{2}\leq 2E{\left(\gamma \left(z\right)\right)}^{2}=2E{\left(\frac{1}{\sigma }\phi \left(\frac{y-f^{*}\left(x\right)}{\sigma }\right)-\frac{1}{\sigma }\phi \left(\frac{y-f(x)}{\sigma }\right)\right)}^{2}\leq \frac{2∥\phi^{\prime }{∥}_{\infty }^{2}}{\sigma^{4}}{∥f-f^{*}∥}_{2}^{2}\leq \frac{2∥\phi^{\prime }{∥}_{\infty }^{2}}{\sigma^{4}}\frac{\Delta_{-}^{2}}{\gamma^{2}}, \end{array}$$

and:

(10) $$\begin{array}{ccc} \eta_{n} & = & \underset{\gamma \in \Gamma }{\sup}{∥\gamma ∥}_{\infty }\leq \frac{∥\phi^{\prime }{∥}_{\infty }}{\sigma^{2}}∥f^{*}{-f∥}_{\infty }\leq \frac{∥\phi^{\prime }{∥}_{\infty }}{\sigma^{2}}{∥f^{*}-f∥}_{K}\leq \frac{∥\phi^{\prime }{∥}_{\infty }}{\sigma^{2}}\frac{\Delta_{+}}{\gamma_{n}^{2}}. \end{array}$$

Combining (7)–(10), we obtain with confidence at least 1−exp{−t}

$$\zeta \leq 2E\zeta +\frac{2∥\phi^{\prime }{∥}_{\infty }}{\gamma_{n}\sigma^{2}}\sqrt{\frac{t}{n}}+\frac{\kappa ∥\phi^{\prime }{∥}_{\infty }\Delta_{+}}{\sigma^{2}\gamma_{n}^{2}}\frac{1+t}{n}.$$

By a symmetrization technique in [42], we have:

$$E\zeta \leq 2ER_{n}\left(\Gamma \right)\leq \frac{2∥\phi^{\prime }{∥}_{\infty }}{\sigma^{2}}ER_{n}\left(F-f^{*}\right).$$

Applying Lemma 3 for $R_{n}\left(F-f^{*}\right)$, we obtain that:

$$E\left[R_{n}\left(F-f^{*}\right)\right]\leq R_{n}\left(F-f^{*}\right)+4\frac{\Delta_{-}}{\gamma_{n}}\sqrt{\frac{2t}{n}}+\frac{\Delta_{+}}{\gamma_{n}^{2}}\frac{1+t}{n},$$

with probability at least 1−2exp{−t}. Moreover, with probability at least 1−2exp{−t}, it holds:

$$\begin{array}{ccc} \zeta & \leq & \frac{8∥\phi^{\prime }{∥}_{\infty }}{\sigma^{2}}R_{n}\left(F-f^{*}\right)+\frac{6∥\phi^{\prime }{∥}_{\infty }\Delta_{-}}{\gamma_{n}\sigma^{2}}\sqrt{\frac{t}{n}}+\frac{5∥\phi^{\prime }{∥}_{\infty }\Delta_{+}}{\gamma_{n}^{2}\sigma^{2}}\frac{1+t}{n}\\ & \leq & \frac{8∥\phi^{\prime }{∥}_{\infty }}{\sigma^{2}}\sum_{j=1}^{p}R_{n}\left(H_{j}-f_{j}^{*}\right)+\frac{6∥\phi^{\prime }{∥}_{\infty }\Delta_{-}}{\gamma_{n}\sigma^{2}}\sqrt{\frac{t}{n}}+\frac{5∥\phi^{\prime }{∥}_{\infty }\Delta_{+}}{\gamma_{n}^{2}\sigma^{2}}\frac{1+t}{n}. \end{array}$$

For the event θ(t), Lemma 1 demonstrates that:

$$|R_{n}\left(f\right)|\leq c_{\alpha }\eta \left(t\right)\xi_{n}{(∥f∥}_{2}+\lambda^{\frac{1}{2}}{∥f∥}_{K}),\forall f\in H_{K},\forall \lambda >0,$$

with confidence 1−exp{−t}. Then,

$$\begin{array}{ccc} \zeta & \leq & \frac{8∥\phi^{\prime }{∥}_{\infty }c_{\alpha }\eta \left(t\right)\xi_{n}}{\sigma^{2}}\underset{f\in F}{\sup}\{\sum_{j=1}^{p}∥f-f_{j}^{*}{∥}_{2}+\lambda^{\frac{1}{2}}\sum_{j=1}^{p}∥f_{j}-f_{j}^{*}{∥}_{K}\}+\frac{6∥\phi^{\prime }{∥}_{\infty }\Delta_{-}}{\gamma_{n}\sigma^{2}}\sqrt{\frac{t}{n}}+\frac{5∥\phi^{\prime }{∥}_{\infty }\Delta_{+}}{\gamma_{n}^{2}\sigma^{2}}\frac{1+t}{n}. \end{array}$$

Taking $\lambda =n^{-\frac{1}{1+\alpha }}$, we can verify that $c\gamma_{n}\geq \xi_{n}$ and $\xi_{n}\lambda^{\frac{1}{2}}\geq c\gamma_{n}^{2}$. Then,

$$\begin{array}{ccc} \zeta & \leq & \frac{8c_{\alpha }\eta \left(t\right){∥\phi^{\prime }∥}_{\infty }}{\sigma^{2}}\left(\Delta_{+}+\Delta_{-}\right)+\frac{6\Delta_{-}{∥\phi^{\prime }∥}_{\infty }}{\sigma^{2}}\sqrt{\frac{t}{A\log\tilde{p}}}+\frac{5\Delta_{+}{∥\phi^{\prime }∥}_{\infty }t}{\sigma^{2}A\log\tilde{p}}, \end{array}$$

for some event $\theta (\Delta_{-},\Delta_{+})$.

For $t=2\log\left(2\sqrt{3}/\log2\right)+A\log\tilde{p}+2\log\tilde{p}$, we deduce that $e^{-\tilde{p}}\leq \Delta_{-}\leq e^{\tilde{p}}$ and $e^{-\tilde{p}}\leq \Delta_{+}\leq e^{\tilde{p}}$ considering ${\left(2\tilde{p}+1\right)}^{2}$ different discrete pairs $\Delta_{-}^{j}=\Delta_{+}^{j}:=2^{-j},j=-\tilde{p},\ldots ,\tilde{p}$, and we deduce that:

$$\begin{array}{ccc} P(\underset{k,j}{⋂}\theta \left(\Delta_{-}^{j},\Delta_{+}^{j}\right)) & \leq & 1-3({\frac{2}{\log2}}^{2}{\tilde{p}}^{2}\exp\{-2\log\left(\frac{2\sqrt{3}}{\log2}-A\log\tilde{p}-2\log\tilde{p}\right\}\leq 1-{\tilde{p}}^{-A}. \end{array}$$

When $\Delta_{-}\leq e^{-\tilde{p}}$ or $\Delta_{+}\leq e^{-\tilde{p}}$, it is trivial to obtain the desired result. □

The proof of Lemma 4 is derived from the proof of Proposition 1 in [28] for the quantile regression. We state our main result on the error bound.

**Theorem** **1.** *Let the regularization parameters of $\hat{f}$ defined in (6) be $\lambda_{1}=\sqrt{\xi }\gamma_{n}$ and $\lambda_{2}=\xi \gamma_{n}^{2}$, where $\xi =\max\{2c\eta \left(t_{0}\right),4\}$ with $\eta \left(t\right)=\max\left\{1,\sqrt{t},t/\sqrt{n}\right\}$, $t_{0}=2\log\left(2\sqrt{3}/\log2\right)+A\log\tilde{p}+2\log\tilde{p}$, and $\gamma_{n}≍\max\left(\sqrt{\frac{A\log\tilde{p}}{n}},{\left(\frac{1}{n}\right)}^{\frac{1}{2(1+\alpha )}}\right)$. Under the conditions of Assumptions 1 and 2, for any $\tilde{p}\geq p$ such that $\log p\leq \sqrt{n}$ and $\log\tilde{p}\geq 2\log\log n$, then for some constant A≥2, such that with probability at least $1-2{\tilde{d}}^{-A}$:*

$$\begin{array}{ccc} R\left(f^{*}\right)-R\left(\hat{f}\right) & \leq & cs∥\phi^{\prime }{∥}_{\infty }\eta \left(t_{0}\right){\left(\eta \left(t_{0}\right)\right)}^{\frac{1}{4}}\sqrt{\gamma_{n}}\leq c{\left(\eta \left(t_{0}\right)\right)}^{\frac{5}{4}}\max\left\{{\left(\frac{A\log\tilde{p}}{c}\right)}^{\frac{1}{4}},{\left(\frac{1}{n}\right)}^{\frac{1}{4(1+\alpha )}}\right\}\\ & \leq & c\max{\left\{\sqrt{A\log\tilde{p}},\frac{A\log\tilde{p}}{\sqrt{n}}\right\}}^{\frac{5}{4}}\cdot \max\left\{{\left(\frac{A\log\tilde{p}}{n}\right)}^{\frac{1}{4}},{\left(\frac{1}{n}\right)}^{\frac{1}{4+4\alpha }}\right\}\\ & \leq & c\max\{\frac{{\left(A\log\tilde{p}\right)}^{\frac{7}{8}}}{n^{\frac{1}{4}}},\frac{{\left(A\log\tilde{p}\right)}^{\frac{1}{2}}}{n^{\frac{1}{4+4\alpha }}},\frac{{\left(A\log\tilde{p}\right)}^{\frac{3}{2}}}{n^{\frac{3}{4}}},\frac{{\left(A\log\tilde{p}\right)}^{\frac{5}{4}}}{n^{\frac{3+2\alpha }{4+4\alpha }}}\}. \end{array}$$

**Proof.**  By the definition of $\hat{f}$ in (6), we know that:

$$\begin{array}{cc} & {\hat{R}}^{\sigma }\left(\hat{f}\right)-\lambda_{1}\sum_{j=1}^{p}∥{\hat{f}}_{j}{∥}_{n}-\lambda_{2}\sum_{j=1}^{p}∥{\hat{f}}_{j}{∥}_{K_{j}}\geq {\hat{R}}^{\sigma }\left(f^{*}\right)-\lambda_{1}\sum_{j=1}^{p}∥f_{j}^{*}{∥}_{n}-\lambda_{2}\sum_{j=1}^{p}{∥f_{j}^{*}∥}_{K_{j}}. \end{array}$$

This implies that:

$$\begin{array}{ccc} & & {\hat{R}}^{\sigma }\left(\hat{f}\right)-R^{\sigma }\left(f^{*}\right)-\lambda_{1}\sum_{j=1}^{p}∥{\hat{f}}_{j}{∥}_{n}-\lambda_{2}\sum_{j=1}^{p}{∥{\hat{f}}_{j}∥}_{K_{j}}\\ & \geq & \left[R^{\sigma }\left(\hat{f}\right)-R^{\sigma }\left(f^{*}\right)\right]-\left[{\hat{R}}^{\sigma }\left(\hat{f}\right)-{\hat{R}}^{\sigma }\left(f^{*}\right)\right]-\lambda_{1}\sum_{j=1}^{p}∥f_{j}^{*}{∥}_{n}-\lambda_{2}\sum_{j=1}^{p}{∥f_{j}^{*}∥}_{K_{j}}. \end{array}$$

Moreover,

(11) $$\begin{array}{ccc} & & R^{\sigma }\left(f^{*}\right)-R^{\sigma }\left(\hat{f}\right)\leq R^{\sigma }\left(f^{*}\right)-R^{\sigma }\left(\hat{f}\right)+\lambda_{1}\sum_{j\notin S}∥{\hat{f}}_{j}{∥}_{n}+\lambda_{2}\sum_{j\notin S}{∥{\hat{f}}_{j}∥}_{K}\\ & \leq & \left[R^{\sigma }\left(f^{*}\right)-R^{\sigma }\left(\hat{f}\right)\right]-\left[{\hat{R}}^{\sigma }\left(f^{*}\right)-{\hat{R}}^{\sigma }\left(\hat{f}\right)\right]+\lambda_{1}\sum_{j\in S}(∥f_{j}^{*}{∥}_{n}-∥{\hat{f}}_{j}{∥}_{n})+\lambda_{2}\sum_{j\in S}(∥f_{j}^{*}{∥}_{K}-∥{\hat{f}}_{j}{∥}_{K})\\ & \leq & \left[R^{\sigma }\left(f^{*}\right)-R^{\sigma }\left(\hat{f}\right)\right]-\left[{\hat{R}}^{\sigma }\left(f^{*}\right)-{\hat{R}}^{\sigma }\left(\hat{f}\right)\right]+\lambda_{1}\sum_{j\in S}∥{\hat{f}}_{j}-f_{j}^{*}{∥}_{n}+\lambda_{2}\sum_{j\in S}{∥{\hat{f}}_{j}-f_{j}^{*}∥}_{K}. \end{array}$$

Taking $\lambda_{1}=\sqrt{\xi }\gamma_{n}$, $\lambda_{2}=\xi \gamma_{n}^{2}$ with $\gamma_{n}=\max\left\{\sqrt{\frac{A\log\tilde{p}}{n}},{\left(\frac{1}{n}\right)}^{\frac{1}{2+2\alpha }}\right\}$, α∈(0,1), we deduce that:

$$\begin{array}{c} \gamma_{n}\sum_{j=1}^{p}{∥{\hat{f}}_{j}-f_{j}^{*}∥}_{2}\leq 2p{\left(\frac{1}{n}\right)}^{\frac{1}{2+2\alpha }}\leq 2\tilde{p}\left(\frac{1}{4}\right)\leq e^{\tilde{p}},\forall n\geq 1,\tilde{p}\geq p, \end{array}$$

and:

$$\gamma_{n}^{2}\sum_{j=1}^{p}∥f_{j}-f_{j}^{*}{∥}_{K_{j}}\leq \gamma_{n}\gamma_{n}\sum_{j=1}^{p}{∥\hat{f}-f^{*}∥}_{K_{j}}\leq e^{-\tilde{p}}.$$

Therefore, we verify that $\hat{f}\in F\left(\Delta_{-},\Delta_{+}\right)$ with $\Delta_{-}\leq e^{\tilde{p}}$ and $\Delta_{+}\leq e^{\tilde{p}}$. With the choices $\lambda_{2}=\lambda_{1}^{2}=\xi \gamma_{n}^{2}$, it holds:

$$\lambda_{1}∥{\hat{f}}_{j}-f_{j}^{*}{∥}_{n}+\lambda_{2}{∥{\hat{f}}_{j}-f_{j}^{*}∥}_{K}\leq 2\left(\lambda_{1}+\lambda_{2}\right)=4\sqrt{\xi }\gamma_{n},j\in S.$$

due to the fact $∥f_{j}{∥}_{n}\leq {∥f_{j}∥}_{K}\leq 1$, for any $f_{j}\in H_{K_{j}}$.

According to Lemma 4 and (11), we obtain:

$$\begin{array}{ccc} & & R^{\sigma }\left(f^{*}\right)-R^{\sigma }\left(\hat{f}\right)\\ & \leq & \frac{c\eta t_{0}{∥\phi^{\prime }∥}_{\infty }}{\sigma^{2}}(\gamma_{n}\sum_{j=1}^{p}∥{\hat{f}}_{j}-f_{j}^{*}{∥}_{2}+\gamma_{n}^{2}\sum_{j=1}^{p}∥{\hat{f}}_{j}-f_{j}^{*}{∥}_{K})+\lambda_{1}\sum_{j\in S}∥{\hat{f}}_{j}-f_{j}^{*}{∥}_{n}+\lambda_{2}\sum_{j\in S}{∥{\hat{f}}_{j}-f_{j}^{*}∥}_{K}+e^{-\tilde{p}}\\ & \leq & \frac{c\eta \left(t_{0}\right){∥\phi^{\prime }∥}_{\infty }}{\sigma^{2}}\sqrt{\xi }\gamma_{n}+e^{-\tilde{p}}, \end{array}$$

with probability at least $1-2{\tilde{p}}^{-A}$.

Notice that $\log\tilde{p}\geq 2\log\log n$ implies that $e^{-\tilde{p}}\leq n^{-2}\leq \gamma_{n}$. Then:

$$R^{\sigma }\left(f^{*}\right)-R\left(\hat{f}\right)\leq \frac{c\eta \left(t_{0}\right){∥\phi^{\prime }∥}_{\infty }}{\sigma^{2}}\sqrt{\xi }\gamma_{n}.$$

Combining this with Theorem 9 in [17] and setting $\sigma =(∥\phi^{\prime }{{∥}_{\infty }\eta \left(t_{0}\right)\sqrt{\xi }\gamma_{n})}^{\frac{1}{4}}$, we obtain the desired result. □

The proof of Theorem 1 is inspired by that of Theorem 1 in [28]; see [28] for more details. According to Theorem 1, we can conclude that the mode-based SpAM can achieve the learning rate with polynomial decay $O(n^{-\frac{1}{4+4\alpha }})$ since ϵ∈[0,1] and $A,\tilde{p}$ are positive constants.

## 4. Experimental Evaluation

**Figure 1:**
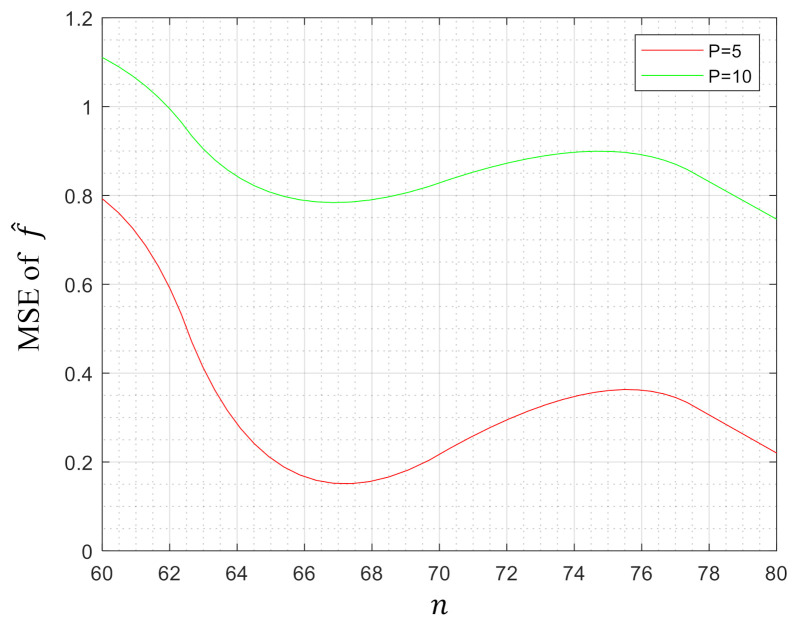
MSE of $\hat{f}$ when $\left(\lambda_{1},\lambda_{2}\right)=\left(0,1\right)$.

**Figure 2:**
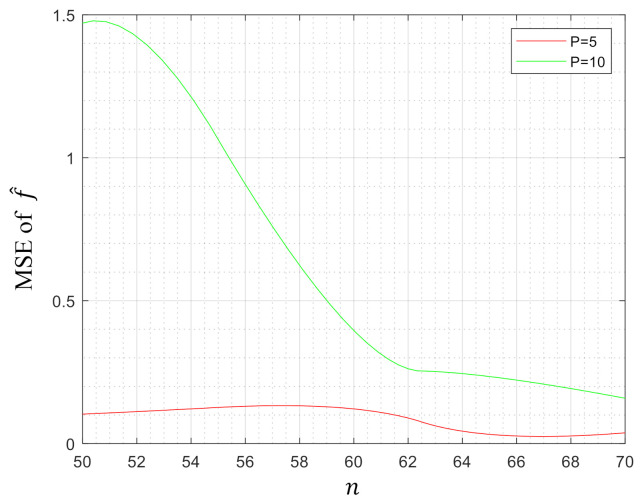
MSE of $\hat{f}$ when $\left(\lambda_{1},\lambda_{2}\right)=\left(1,0\right)$.

**Figure 3:**
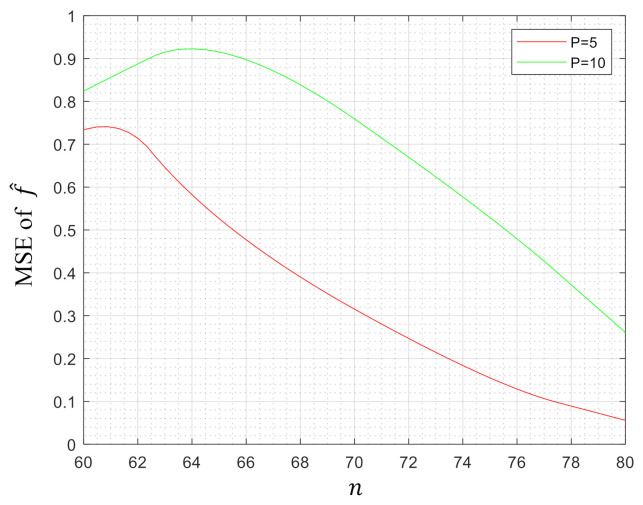
MSE of $\hat{f}$ when $\left(\lambda_{1},\lambda_{2}\right)=\left(1,1\right)$.

To demonstrate the efficiency of our method, in this section, we evaluated our model on some synthetic datasets. The data in $R^{p}$ with dimension p=5 and p=10 were generated randomly according to the uniform distribution on the interval [0,1]. Then, we computed the MSE of our estimator $\hat{f}$. Figure 1, Figure 2 and Figure 3 depict the MSE of $\hat{f}$ when the parameter pair $\left(\lambda_{1},\lambda_{2}\right)=\left(0,1\right),\left(1,0\right)$ and (1,1), respectively, while the number of samples *n* varies from 50/60 to 80/90. This paper used Yalmip [43] modeling in the MATLAB environment and called *fmincon* to solve the problem. From the figures, we can notice that the MSEs tended to decrease with the increase of the number of samples *n* under three kinds of parameter settings, which verified that our method was effective in the regression of high-dimensional data.

## 5. Conclusions

In this work, we proposed a mode-based sparse additive model and established its generalization error bound. The theoretical results extended the previous mean-based analysis to the mode-based approach. We demonstrated that the mode-based SpAM can achieve the learning rate with polynomial decay $O(n^{-\frac{1}{4+4\alpha }})$, which is comparable to the previous result in [15] with $O(n^{-\frac{1}{7}})$. In the future, it will be important to further explore the variable selection consistency of the proposed model.

## Data Availability

The synthetic data generation method of the simulation experiment has been introduced in the experimental part.
